# Co-option of immune and digestive cellular machinery to support photosymbiosis in amoebocytes of the upside-down jellyfish *Cassiopea xamachana*

**DOI:** 10.1242/jeb.249849

**Published:** 2025-05-12

**Authors:** Angus B. Thies, Maitri Rangarajan-Paul, Daniel Wangpraseurt, Martin Tresguerres

**Affiliations:** Scripps Institution of Oceanography, University of California, San Diego, La Jolla, CA 92093, USA

**Keywords:** V-ATPase, Proton pump, Symbiosome, Symbiosis, Digestion, Carbon concentrating mechanism

## Abstract

The upside-down jellyfish *Cassiopea* spp. host their algal symbionts inside a subset of amoebocytes, phagocytic cells that also play innate immune functions akin to macrophages from vertebrate animals. Amoebocyte precursors phagocytose algae from the jellyfish gut and store them inside intracellular compartments called symbiosomes. Subsequently, the precursors migrate to the mesoglea, differentiate into symbiotic amoebocytes, and roam throughout the jellyfish body, where the algae remain photosynthetically active and supply the jellyfish host with a significant portion of their organic carbon needs. Here, we show that the amoebocyte symbiosome membrane contains V-H^+^-ATPase (VHA), the proton pump that acidifies phagosomes and lysosomes in all eukaryotes. Many symbiotic amoebocytes also abundantly express a carbonic anhydrase (CA), an enzyme that reversibly hydrates CO_2_ into H^+^ and HCO_3_^−^. Moreover, we found that the symbiosome lumen is pronouncedly acidic and that pharmacological inhibition of VHA or CA activities significantly decreases photosynthetic oxygen production in live jellyfish. These results point to a carbon concentrating mechanism (CCM) that co-opts VHA and CA from the phago-lysosomal machinery that ubiquitously mediates food digestion and innate immune responses. Analogous VHA-dependent CCMs have been previously described in reef-building corals, anemones and giant clams; however, these other two cnidarians host their dinoflagellate algae inside gastrodermal cells – not in amoebocytes – and the clam hosts theirs within the gut lumen. Thus, our study identifies an example of convergent evolution at the cellular level that might broadly apply to invertebrate–microbe photosymbioses while also providing evolutionary links with intracellular and extracellular food digestion and the immune system.

## Teaser

**Summary:** Jellyfish amoebocytes promote photosynthesis by their endosymbiotic algae using proton pumps and carbonic anhydrases. These two enzymes ubiquitously acidify phagosomes, lysosomes and invertebrate guts, establishing functional and evolutionary links between symbiosis, food digestion and immunity.

## INTRODUCTION

Non-bilateral invertebrates including cnidarians (coral, anemone, jellyfish and relatives) largely rely on phagocytosis for feeding and for defense against bacterial, fungal and algal pathogens (Steinmetz, 2019). The vesicular- or vacuolar-type H^+^-ATPase (VHA) is a proton pump enzyme that plays an essential role in this process by acidifying phagosomes, lysosomes and phago-lysosomes (reviewed in Freeman et al., 2022). VHA activity in these organelles provides a corrosive microenvironment, activates hydrolytic enzymes that further break down the engulfed material, and energizes the transport of nutrients into the cytosol (Kinchen and Ravichandran, 2008).

Phagocytosis is also the initial step in the establishment of intracellular endosymbiosis between cnidarians and dinoflagellate algae of the family Symbiodiniaceae (reviewed in Trench, 1979). After phagocytizing free-living algae, the animal cell sequesters them in an arrested phagosome known as the ‘symbiosome’ (reviewed in Davy et al., 2012). The algae remain photosynthetically active and translocate sugars, lipids, amino acids and other photosynthates to the host, which in many cases satisfies the majority of the animal's nutritional demand (Verde and McCloskey, 1998; Tanaka et al., 2018). In addition, algal photosynthesis produces O_2_ that favors aerobic respiration by the holobiont; indeed, the aerobic respiration rate of photosymbiotic invertebrates is several-fold higher during the day (‘light respiration’) than at night (‘dark respiration’) (Armstrong et al., 2018; Kühl et al., 1995; Linsmayer et al., 2024; Schrameyer et al., 2014; Shick, 1990). In turn, the animal host generates CO_2_ by aerobic respiration, which fuels photosynthesis by their symbionts (Muscatine et al., 1989) and provides them with ammonia and other essential micronutrients (reviewed in Davy et al., 2012). These metabolic exchanges afford both symbiotic partners an advantage in competitive environments.

The majority of molecular and cellular studies about cnidarian symbiosis have focused on anthozoans (corals and anemones), which host their algal symbionts inside gastrodermal cells called ‘symbiocytes’ (Tresguerres et al., 2017). These studies have revealed that symbiosomes share protein machinery and biochemical conditions with phago-lysosomes, thus providing further support for the phagocytotic origin of cnidarian photosymbioses. The pH inside their symbiosomes is <6 and as low as 4 (Venn et al., 2009; Barott et al., 2015), which is at least 10-fold more acidic than the host cell cytosol (pH ∼7.1–7.6) (Venn et al., 2009; Barott et al., 2017; Fountain et al., 2021) and 100-fold more acidic than seawater (pH ∼8.0). Similar to phago-lysosomes of professional phagocytic cells, this acidification is generated, at least in part, by VHAs present in the symbiosome membrane (Barott et al., 2015, 2022; Tresguerres, 2016; Tresguerres et al., 2020). In phagosomes, a similar acidic pH gradient promotes the degradation of phagocytosed materials and drives secondary metabolite transport into the cytosol (reviewed in Kinchen and Ravichandran, 2008). In the symbiosome of anthozoans, VHA activity has been additionally co-opted to concentrate dissolved inorganic carbon (DIC) in the microenvironment surrounding the symbiotic algae and promote photosynthesis (Barott et al., 2015, 2022). This carbon concentrating mechanism (CCM) presumably helps overcome the low affinity of algal Rubisco for CO_2_, which precludes efficient carbon fixation at ambient *P*_CO_2__ levels and is exacerbated by light-limiting, low DIC environments (Leggat et al., 2002; Yee et al., 2023). Another proposed component of anthozoan CCMs are carbonic anhydrase (CA) enzymes from the animal that reversibly catalyze the hydration and dehydration of CO_2_ into H^+^ and HCO_3_^−^. Cnidarian host cells may have one CA isoform on their plasma membrane facing the gastric cavity and another isoform in the cytoplasm in close association with the symbiosome membrane (Al-Moghrabi et al., 1996; Benazet-Tambutte et al., 1996; Weis, 1991, 1993; Weis et al., 1989).

Endosymbiosis in scyphozoan jellyfish has evolved independently from anthozoans (Kayal et al., 2018), and instead of hosting symbiotic algae inside symbiocytes, jellyfish host theirs inside motile phagocytic cells called ‘amoebocytes’ (reviewed in Djeghri et al., 2019). Like symbiocytes, amoebocytes derive from endodermal cells and originally line up the gastric cavity of the polyp. However, after they phagocytose one or multiple algae, the emerging symbiosomes move to the basal pole of the cell, which then detaches from the gastrodermis and migrates into the mesoglea (the connective tissue between the epidermis with the gastrodermis) (Colley and Trench, 1985; Fitt and Trench, 1983). In the mesoglea, the cell differentiates into an amoebocyte hosting endosymbiotic algae that, like the symbiocytes in anthozoans, supply abundant photosynthates that can exceed the organic carbon requirements of the host (Welsh et al., 2009). However, amoebocytes have two important differences compared with symbiocytes: (1) each amoebocyte can host 10 or more symbiotic algae (Fitt et al., 2021) whereas each symbiocyte typically contains only one, and occasionally two or three (Venn et al., 2009), and (2) amoebocytes have been suggested to roam the greatly enlarged mesoglea of jellyfish, unlike symbiocytes, which are attached to the gastrodermis of anthozoans. This motility allows amoebocytes to regulate the delivery of photosynthates to specific non-symbiotic cells throughout the animal including muscle, gastric or nervous cells (Lyndby et al., 2020). Additionally, polyps of the upside-down jellyfish *Cassiopea* spp. will not develop into medusae until an appropriate density of amoebocyte-bound, photosynthetically active symbionts is achieved (Hofmann et al., 1978; Jinkerson et al., 2022), and therefore endosymbiosis is critical to both the energy budget and life cycle of *Cassiopea*.

Here, we explored whether the amoebocytes of *C. xamachana* employ a similar CCM to that of anthozoan symbiocytes despite their independent evolutionary origin and stark differences in development, morphology and motility. We found that amoebocytes contain both VHA and CA at high abundance and closely associated with the symbiosome, and that VHA and CA activities enhance photosynthetic O_2_ production. The results indicate independently evolved but mechanistically conserved CCMs in amoebocytes and symbiocytes, establishing links between phagocytosis, food digestion, innate immunity and endosymbiosis that may broadly apply to invertebrate–microbe symbioses.

## MATERIALS AND METHODS

### Animals

*Cassiopea xamachana* Bigelow 1892 medusae (mixed assemblage of strains T2C, JB2, JB4 and JB8) were maintained in a recirculating 100 liter aquarium filled with artificial seawater with the following conditions: temperature ∼25–26°C, salinity ∼36 ppt, alkalinity ∼2.4 meq l^−1^, pH ∼8.1–8.2, nitrate 0 ppm, nitrite 0 ppm, total ammonia <0.5 mg l^−1^ and calcium ∼400 ppm. The aquarium was illuminated on a 12 h:12 h light:dark cycle at a downwelling irradiance of 250 μmol photons m^−2^ s^−1^ (400–700 nm) using LED panels (Hydra 64 HD, Aqua Illumination, Bethlehem, PA, USA) as determined using an MQ-510 full-spectrum quantum sensor (Apogee Instruments, Logan, UT, USA). pH and temperature were monitored using a HACH PHC101pH Electrode (HACH, Loveland, CO, USA). Total ammonia, nitrate and nitrite were monitored using API Water Test Kits (Mars Fishcare, Chalfont, PA, USA). Calcium and alkalinity levels were measured using marine calcium and alkalinity meters (Hanna Instruments, Woonsocket, RI, USA). Animals were fed daily with either freshly hatched *Artemia* (San Francisco Strain, Brine Shrimp Direct, Ogden, UT, USA) or a 50:50 mixture of PhytoFeast and OysterFeast (Reef Nutrition, Campbell, CA, USA).

### Antibodies

Tissues were probed for VHA using a custom rabbit polyclonal antibody developed using a peptide antigen matching a conserved region of VHA's B subunit (VHA_B_; AREEVPGRRGFPGY; GenScript Biotech Corporation, Piscataway, NJ, USA), which is 100% conserved in species ranging from corals to humans (Armstrong et al., 2018; Barott et al., 2015; Damsgaard et al., 2020; Roa and Tresguerres, 2016; Tresguerres et al., 2013). Antibodies against human CAII were purchased from Rockland (product 100-401-136, lot 20721, Gilbertsville, PA, USA) and have been previously used to detect CA in mammals, fishes and *Osedax* worms (Perry et al., 2010; Tresguerres et al., 2013; Yasukawa et al., 2007).

### Western blotting

**Fig. 1.**
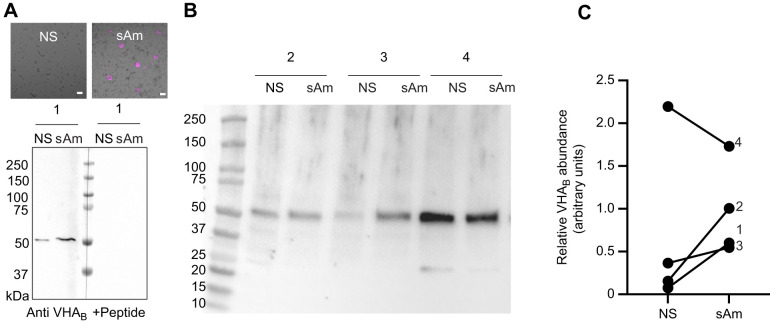
**V-H^+^-ATPase (VHA) abundance in *Cassiopea xamachana*.** (A) Western blot showing VHA_B­­_ immunoreactive bands in tissue fractions enriched for non-symbiotic (NS) cells and symbiotic amoebocytes (sAm) and peptide-preabsorption control (+peptide). (B) Western blot against VHA_B_ in NS and sAm from three additional jellyfish. (C) Quantification of VHA_B_ abundance in the four jellyfish (1–4). There were no statistically significant differences between NS and sAm (paired *t*-test).

A ∼1 cm fragment of *C. xamachana* bell tissue was flash-frozen in liquid nitrogen and powdered using a mortar and pestle. Powdered tissue was homogenized in S22 buffer (450 mmol l^−1^ NaCl, 10 mmol l^−1^ KCl, 58 mmol l^−1^ MgCl_2_, 10 mmol l^−1^ CaCl_2_, 100 mmol l^−1^ Hepes, pH 7.80; Sigma-Aldrich, St Louis, MO, USA) supplemented with protease inhibitor cocktail (P2714; Sigma-Aldrich) and phosphatase inhibitors (PhosStop; Roche Applied Science, Penzberg, Germany) with a glass homogenizer on ice. Cell debris was pelleted by 100×rcf centrifugation (4 min, 4°C). Supernatant was saved and subjected to a 3000×rcf centrifugation (4 min, 4°C) to generate a supernatant fraction enriched in epidermal and non-symbiotic gastrodermal cells and a pellet fraction enriched in amoebocytes containing symbiotic algae (modified from a protocol to enrich for coral symbiocytes; Barott et al., 2022). Enrichment of symbiotic amoebocytes in the pellet was confirmed by visualization of aliquots in the microscope (see Fig. 1 for an example). The pellet was resuspended in 100 µl homogenization buffer. All samples were sonicated for 3×30 s bursts with 1 min rests on ice. Protein concentrations in both fractions were measured using a Bradford assay with a bovine serum albumin standard curve (Bio-Rad, Hercules, CA, USA). Homogenates were then incubated in Laemmli sample buffer with 5% (v/v) β-mercaptoethanol for 5 min at 95°C (VHA_B_) or 15 min at 70°C (CA) and equal protein of each fraction was loaded on a sodium dodecyl sulphate–polyacrylamide gel electrophoresis gel. Proteins were separated at 60 V for 30 min followed by 200 V for 1 h in Tris-glycine running buffer [25 mmol l^−1^ Tris-base, 192 mmol l^−1^ glycine, 0.1% (w/v) sodium dodecyl sulphate, pH 8.3; Sigma-Aldrich]. Following electrophoresis, proteins were transferred from the gel onto a polyvinylidene difluoride (PVDF) membrane using a Mini Trans-Blot Cell (Bio-Rad) filled with Towbin transfer buffer [25 mmol l^−1^ Tris, 192 mmol l^−1^ glycine, 20% (v/v) methanol, pH 8.3; Sigma-Aldrich] overnight. The membrane was washed for 5 min in Tris-buffered saline with 0.1% Tween (TBS-T) on an orbital shaker at room temperature to remove transfer buffer. The membrane was blocked for 1 h on an orbital shaker in blocking buffer [5% (w/v) fat-free milk powder in TBS-T] at room temperature and then incubated overnight at 4°C with either 0.24 µg ml^−1^ polyclonal anti-VHA_B_ primary antibody, anti-VHA_B_ primary antibody with 60× excess peptide on a molar basis (‘preabsorption control’) or 0.9 µg ml^−1^ polyclonal anti-CA primary antibody in blocking buffer. The membrane was washed with 3×10 min TBS-T washes before incubation with secondary antibody (goat anti–rabbit–horseradish peroxidase diluted 1:5000; Bio-Rad) for 1 h on a shaker at room temperature. Membranes were again washed with 3×10 min TBS-T washes. Bands were visualized using Clarity Max Western ECL Substrate (Bio-Rad) and imaged using a Chemidoc Imaging system (Bio-Rad). Protein sequences were sourced from the draft *C. xamachana* genome available at https://mycocosm.jgi.doe.gov/Casxa1/Casxa1.home.html (Ohdera et al., 2019): VHA_B_ protein ID 22327, CA protein ID 1670. Prediction of protein domains was performed using InterPro, signal peptides using DeepLoc 2.0 and N-glycosylation sites using NetNGlyc 1.0 (Gupta and Brunak, 2002; Paysan-Lafosse et al., 2023; Thumuluri et al., 2022).

### Immunohistochemistry (IHC) and LysoSensor imaging

Whole *C. xamachana* were fixed in 3% paraformaldehyde in S22 buffer overnight at 4°C on an orbital shaker. Tissues were dehydrated, embedded in paraffin wax, sectioned at 10 µm and rehydrated following previously described protocols for corals (Barott and Tresguerres, 2015). Isolated cells were prepared by submerging ∼1 cm bell slices in 0.22 μm filtered seawater (FSW) and homogenized into a slurry using a razor blade and gentle pipetting. Cells were pelleted by centrifugation (3000×rcf, 4 min, 23°C) and resuspended in the same fixative as tissues on a rotator (15 min, 4°C). Cells were pelleted (3000×rcf, 4 min, 4°C), washed in S22 buffer (three washes) and spread onto glass slides to air-dry for 25 min. Rehydrated tissue sections and isolated cells were permeabilized in 0.2% Triton-X (v/v) in phosphate buffered saline (PBS-TX) and incubated in blocking buffer (2% normal goat serum and 0.5% keyhole limpet hemocyanin in PBS-TX) for 1 h at room temperature. Slides were incubated overnight (4°C) with either 0.962 µg ml^−1^ polyclonal anti-VHA_B_ primary antibody in blocking buffer, anti-VHA_B_ primary antibody with 13× excess peptide on a molar basis (‘preabsorption control’) in blocking buffer, 2.45 µg ml^−1^ polyclonal anti-CA primary antibody in blocking buffer or blocking buffer alone. Sections were washed with 3×5 min PBS-TX washes before incubation with 4 µg ml^−1^ secondary antibody (goat anti–rabbit–Alexa Fluor 488; A-11008; Invitrogen, Carlsbad, CA, USA) in blocking buffer for 1 h at room temperature followed by 1 µg ml^–1^ 4′,6-diamidino-2-phenylindole (DAPI) DNA stain (Invitrogen) for 5 min. Slides were washed with 3×5 min PBS-TX to remove unbound secondary antibodies and DAPI before mounting with ProLong Glass Antifade Mountant (P36982; Invitrogen). *In vivo* assessment of symbiosome acidity was achieved by incubating freshly isolated amoebocytes (see above) with 1 μmol l^−1^ LysoSensor Green D-189 (LSG; L7535; Invitrogen) in FSW for 1.5 h (250 μmol photons m^−2^ s^−1^, 25°C). Cells were moved to a poly-d-lysine-coated glass-bottom dish for imaging.

Confocal Airyscan microscopy was performed on a Zeiss AxioObserver Z1 connected to a laser scanner equipped with 405, 488, 561 and 640 nm laser lines (Zeiss LSM 800 with Airyscan, Carl Zeiss AG, Oberkochen, Baden-Württemberg, Germany). This device uses a 32-channel photomultiplier detector and linear deconvolution to obtain 140 nm lateral (*x*–*y*) and 400 nm axial (*z*) resolution (Alexa Fluor 488, excitation: 488 nm, emission: 485–593 nm; LSG, excitation: 488 nm, emission: 400–650 nm; chlorophyll, excitation: 640 nm, emission: 650–700 nm; DAPI, excitation: 405 nm, emission: 400–480 nm). To facilitate visualization by color-blind readers, VHA_B_, CA, LSG, chlorophyll and DAPI signals are presented using the false colors yellow, orange, green, violet and blue, respectively, in all figures.

### Pulse amplitude modulated (PAM) fluorometry

The *in vivo* photosystem efficiency of *C. xamachana* symbionts was measured using a Diving PAM device (Dive PAM II, Heinz Walz GmbH, Germany); data were recorded using the accompanying WinControl software (v3.32). Four animals were held in 100% air-saturated 0.1% (v/v) DMSO FSW (26°C) in darkness (0 μmol photons m^−2^ s^−1^) for 30 min. The maximum quantum yield of photosystem II (PSII) (*F*_v_/*F*_m_) was measured by applying a saturating light pulse (1 s, 470 nm, >27,000 μmol photons m^−2^ s^−1^) for five randomly chosen spots. The effective quantum yield of PSII was determined at five random spots on the same animal following an acclimation period of 5 min at 250 μmol photons m^−2^ s^−1^ provided by an ACE Light Source with a full-spectrum halogen EKE lamp (Schott AG, Mainz, Germany) calibrated with a MQ-510 full-spectrum quantum sensor (Apogee Instruments). This measurement procedure was repeated with 1 μmol l^−1^ concanamycin A (ConcA) FSW, 20 μmol l^−1^ ethoxzolamide (EZ) FSW and 100 μmol l^−1^ 3-(3,4-dichlorophenyl)-1,1-dimethylurea (DCMU) FSW. *F*_v_/*F*_m_ was calculated by: *F*_v_/*F*_m_=(*F*_m_–*F*_0_)/*F*_m_, where *F*_v_ is variable fluorescence, *F*_m_ is maximum fluorescence and *F*_0_ is minimum fluorescence. Effective photochemical quantum yield under illumination [*Y*(II)] was calculated as: *Y*(II)=(*F*_m_–*F*)/*F*_m_, where *F* is the fluorescence level determined immediately before a saturation pulse is applied. Stock solutions of ConcA, EZ and DCMU were prepared so the final DMSO concentration was 0.1% (v/v).

### Oxygen dynamics

Dark respiration (*R*_D_) and net photosynthesis (*P*_N_) for individual *C. xamachana* bell slices (∼1 cm) were measured in sealed glass chambers at 26°C using a Clark-type oxygen microelectrode (500 µm tip, <15 s 90% response time; Unisense, Aarhus, Denmark) that was calibrated with anoxic (bubbling with N_2_ gas) and 100% air-saturated FSW. Experimental chambers were stirred with an internal magnetic stir bar and temperature was maintained via external water bath. Data were recorded using the accompanying SensorTrace software (v3.4.3; Unisense). Light was provided using an ACE Light Source with a full-spectrum halogen EKE lamp (Schott AG) and calibrated with an MQ-510 full-spectrum quantum sensor (Apogee Instruments). *R*_D_ and *P*_N_ were measured under darkness and 250 μmol photons m^−2^ s^−1^, respectively. Prior to the start of all microelectrode trials, bell fragments were immobilized by trimming the outer rhopalium and nerve ring; this halted all rhythmic pulsing of the bell except for occasional twitching.

To test whether DMSO alone affected *R*_D_ or *P*_N_, bell slices were placed in 100% O_2_-saturated FSW in darkness for 15 min before moving into sealed test chambers filled with 100% O_2_-saturated FSW (*n*=5). *R*_D_ was measured for 15 min before illuminating the animal with 250 μmol photons m^−2^ s^−1^ and measuring *P*_N_ for 15 min. The slice was rested for 15 min in 100% air-saturated FSW in darkness before moving into sealed test chambers filled with 100% air-saturated 0.1% (v/v) DMSO FSW. *R*_D_ was measured for 15 min before illuminating the bell slice with 250 μmol photons m^−2^ s^−1^ and measuring *P*_N­_ for 15 min. To ignore O_2_ consumption spikes associated with occasional animal twitching, *R*_D_ and *P*_N_ rates were determined by manually fitting trendlines to the linear portion of the oxygen curves (MATLAB R2022a). Trendlines were fitted in a blind manner: O_2_ curves were displayed without identifiers and a trendline was fitted by an independent observer. Only data from the center of the curves were considered (200–800 s).

To test whether ConcA or EZ affected *R*_D_ or *P*_N_, bell slices were placed in 100% air-saturated FSW in darkness for 15 min before moving into sealed test chambers filled with 100% air-saturated DMSO FSW. *R*_D_ was measured for 15 min before illuminating the slice with 250 μmol photons m^−2^ s^−1^ and measuring *P*_N_ for 15 min. The animal was rested for 15 min in 100% air-saturated FSW in darkness before moving into sealed test chambers filled with 100% air-saturated FSW containing 1 μmol l^−1^ ConcA (*n*=6) or 20 μmol l^−1^ EZ (*n*=6). *R*_D_ was measured for 15 min before illuminating the animal with 250 μmol photons m^−2^ s^−1^ and measuring *P*_N­_ for 15 min. This experiment was repeated with 100% air-saturated FSW containing 100 μmol l^−1^ DCMU as a positive control (*n*=4). Only bell fragments displaying a positive *P*_N­_ rate were included in data analysis. DMSO, ConcA, EZ and DCMU experiments were repeated without any animal tissue (*n*=3) as negative controls demonstrating that neither illumination nor drug additions interacted with the microelectrode. Stock solutions of ConcA, EZ and DCMU were prepared so final DMSO concentrations were 0.1% (v/v) in all trials.

### Statistics

Data were analyzed in GraphPad Prism v10.0.1 (San Diego, CA, USA). Data normality and homogeneity of variance were tested using the Shapiro–Wilk normality test. PAM fluorometry data were analyzed using repeated measures one-way ANOVAs with Dunnett's multiple comparisons tests. Microelectrode blank controls were analyzed with two-way repeated measures ANOVA. *R*_D_ and P_N_ data were analyzed using Wilcoxon matched-pairs signed rank or paired *t*-tests.

## RESULTS

### VHA and CA proteins are highly abundant in amoebocytes and closely associated with symbiosomes

The anti-VHA_B­_ antibodies detected a single band of the expected ∼55 kDa size in western blotting with *C. xamachana* tissues; the epitope is 100% conserved in *C. xamachana* and no signal was present in peptide preabsorption controls (Fig. 1A). In three out of four samples, VHA_B­_ abundance was highest in tissue fractions enriched in symbiotic amoebocytes (Fig. 1A–C). Immunostaining confirmed high abundance of VHA_B_ in symbiont-hosting amoebocytes in both the bell and oral arms (Fig. 1D,E).

**Fig. 2.**
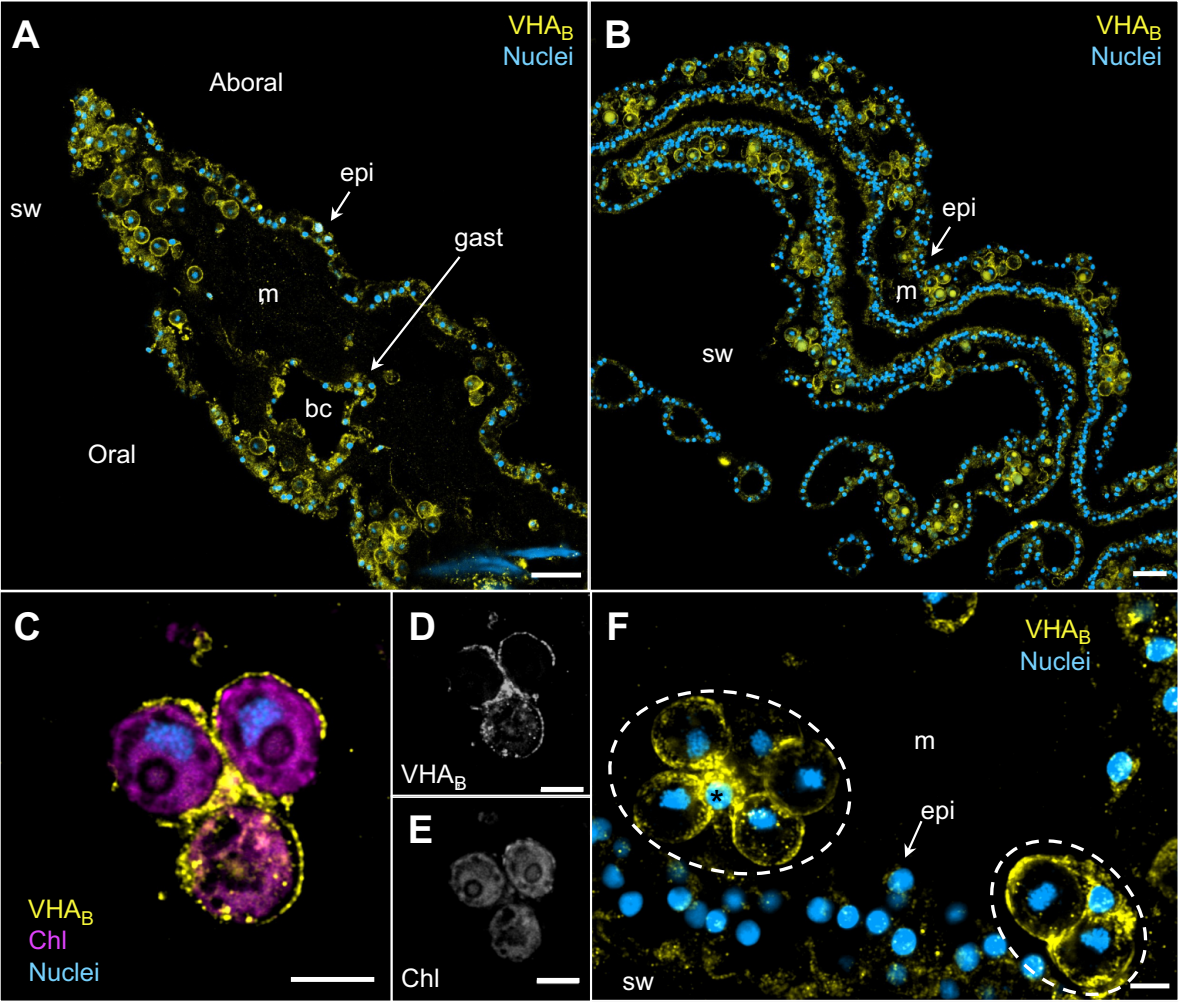
**VHA localization in *C. xamachana*.** VHA_B_ immunolabeling of the (A) bell and (B) oral arms. Both tissues contain abundant symbiotic amoebocytes throughout the mesoglea. Scale bars: 20 µm. (C–E) Airyscan confocal images showing VHA_B_ and chlorophyl (Chl) signals and the corresponding grayscale channels. (F) Airyscan confocal z-stacked image of VHA_B_ immunolabeling of two symbiotic amoebocytes (dashed outlines) containing five and two symbiotic algae, respectively. Scale bars: 5 µm (C–F). bc, brachial canal; epi, epidermis; gast, gastrodermis; m, mesoglea; sw, seawater; *, amoebocyte nucleus.

Confocal microscopy revealed strong VHA_B_ immunofluorescence signal in all symbiotic amoebocytes throughout the jellyfish bell and oral arms and in many epidermal and gastrodermal cells bordering seawater and brachial cavities, respectively (Fig. 2A,B). Further Airyscan confocal ‘super-resolution’ imaging showed the VHA_B_ signal in symbiotic amoebocytes formed a tight ring surrounding each algal symbiont (Fig. 2C). This VHA_B_ immunostaining pattern was observed in every symbiotic amoebocyte regardless of the number of symbiotic algae they hosted (Fig. 2D).

**Fig. 3.**
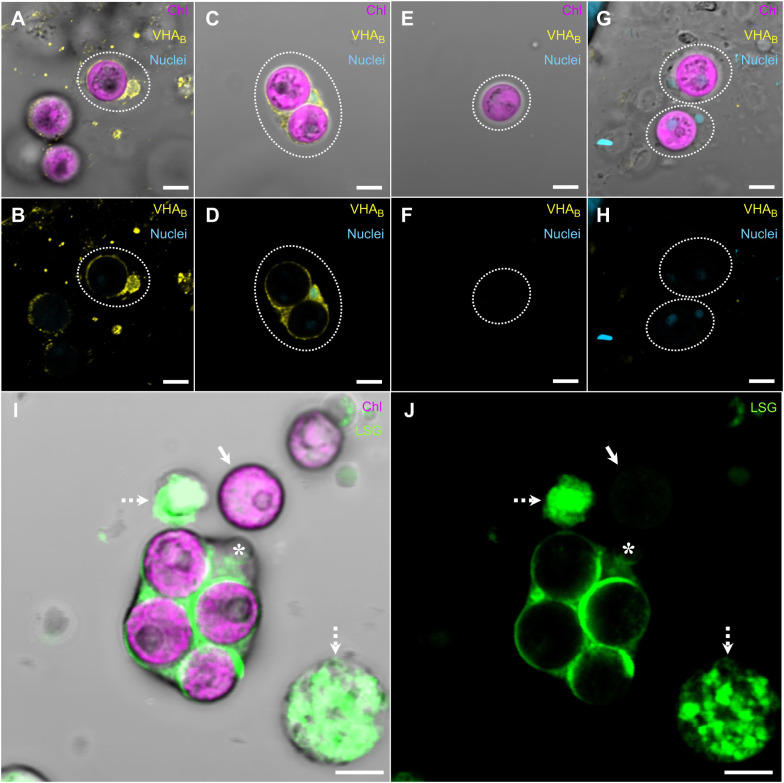
**VHA localization and LysoSensor Green (LSG) accumulation in isolated *C. xamachana* cells.** (A,B) Amoebocyte containing one symbiotic alga. (C,D) Amoebocyte containing two symbiotic algae. (E,F) Free alga that was separated from its host amoebocyte during the isolation process. (G,H) Secondary antibody controls of two amoebocytes hosting one symbiotic alga each. The dotted rings in A–H show corresponding areas between paired panels. (I,J) Amoebocyte labeled with 1 µmol l^−1^ LSG. Panels are paired with and without brightfield differential interference contrast. *, amoebocyte nucleus; arrow, symbiont separated from its host amoebocyte; dotted arrow, non-symbiotic cells. Scale bars: 5 µm.

The close association of VHA with algae-hosting symbiosomes was confirmed by immunocytochemistry of freshly isolated and fixed *C. xamachana* amoebocytes (Fig. 3A–D). In contrast, algal cells that became free during the isolation process did not display VHA_B_ signal (Fig. 3E,F), nor did those incubated without anti-VHA_B_ antibodies (Fig. 3G,H). Labeling of freshly isolated and live amoebocytes with the acidotropic probe LSG revealed that the symbiosome was acidic. LSG consistently labeled every symbiosome as well as other intracellular structures in non-symbiotic cells (Fig. 3I,J).

**Fig. 4.**
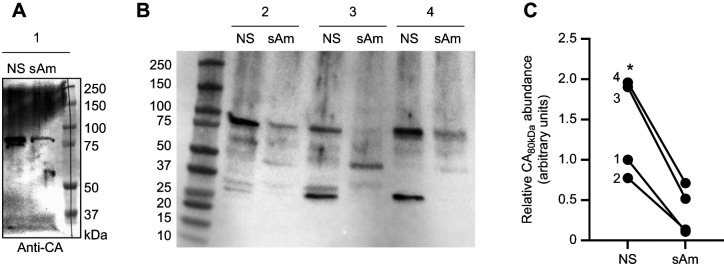
**Carbonic anhydrase (CA) abundance in *C. xamachana*.** (A) Western blot showing 75 and 80 kDa immunoreactive bands in tissue fractions enriched for non-symbiotic (NS) cells and symbiotic amoebocytes (sAm). (B) Western blot against CA showing additional lower molecular weight bands in in NS and sAm from three additional jellyfish. (C) Quantification of 80 kDa band (CA_80kDa_) abundance in the four jellyfish (1–4). The asterisk indicates a statistically significant difference between NS and sAm (paired *t*-test).

Anti-CA antibodies detected western blot protein bands at ∼75 and ∼80 kDa in tissue fractions containing non-symbiotic cells, but only the latter in fractions enriched with symbiotic amoebocytes (Fig. 4A). Further western blot analyses using a shorter gel run time to capture lower molecular weight proteins revealed the abundant presence of ∼30 kDa immunoreactive bands exclusively in tissue fraction lacking symbiotic amoebocytes (Fig. 4B). Quantification of the 80 kDa band showed higher abundance in the non-symbiotic cell fraction compared with the fraction enriched for symbiotic amoebocytes (Fig. 4C).

**Fig. 5.**
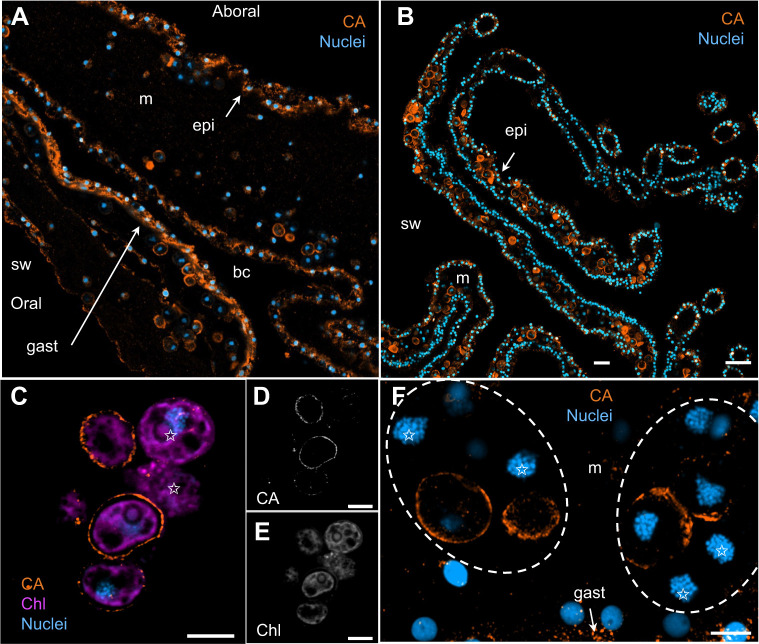
**CA localization in *C. xamachana*.** CA immunolabeling of the (A) bell and (B) oral arms. Both tissues contain abundant amoebocytes throughout the mesoglea. Scale bars: 20 µm. (C–E) Airyscan confocal images showing CA and chlorophyl (Chl) signals and the corresponding grayscale channels. (F) Airyscan confocal z-stacked image of CA immunolabeling of two symbiotic amoebocytes. Detail of the dotted rectangle outlined in A highlighting two amoebocytes containing five and seven symbiotic algae, respectively. White stars denote symbiosomes lacking CA signal. Scale bars: 5 µm (C–F). bc, brachial canal; epi, epidermis; gast, gastrodermis; m, mesoglea; sw, seawater.

These patterns were also seen in immunostained sections showing the most intense CA-like signal in epidermal and gastrodermal cells in both the bell and the arms (Fig. 5A,B). In these cells, the CA-like signal looked cytoplasmic and concentrated near the apical membrane bordering seawater and brachial cavities, respectively. In contrast, the CA-like signal in symbiotic amoebocytes surrounded the periphery of algal symbionts resembling the pattern for VHA_B_ but forming a sharper ring (Fig. 5C–F). And unlike VHA_B_, CA-like signal was not observed in every amoebocyte, which often contained both CA-positive and CA-negative symbiosomes (Fig. 5C–F).

### VHA and CA activities promote symbiont photosynthesis

**Fig. 6.**
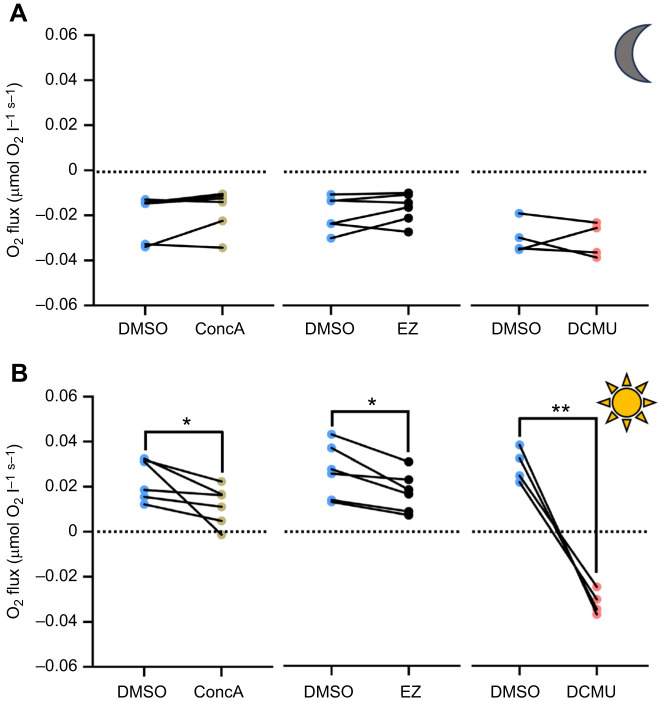
**Effect of VHA, CA and photosystem II (PSII) inhibition on *C. xamachana* dark respiration (*R*_D_) and net photosynthesis (*P*_N_).** Fragments of bell tissue were incubated with DMSO and then with either concanamycin A (ConcA; 1 μmol l^−1^), ethoxzolamide (EZ; 20 μmol l^−1^) or 3-(3,4-dichlorophenyl)-1,1-dimethylurea (DCMU; 100 μmol l^−1^). (A) *R*_D_ was not affected by any drug (Wilcoxon or paired *t*-tests; *P*>0.05). (B) *P*_N_ measured under 250 μmol photons m^−2^ s^−1^. All inhibitors (ConcA, EZ and DCMU) significantly decreased *P*_N_ compared with DMSO alone (paired *t*-tests: *P=0.0445, *P=0.0115 and **P=0.0022, respectively). *N*=4–6.

**Fig. 7.**
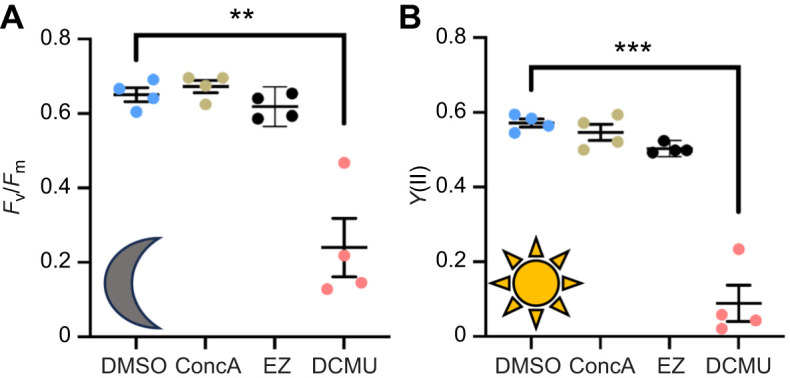
**Effect of VHA, CA and PSII inhibition on PSII efficiency in *C. xamachana*.** (A) Maximal photochemical quantum yield in the dark (*F*_v_/*F*_m_). (B) Effective photochemical quantum yield under 250 μmol photons m^−2^ s^−1^ illumination [*Y*(II)]. Only DCMU had a significant effect on *F*_v_/*F*_m_ and *Y*(II) (repeated measures one-way ANOVA with Dunnett's multiple comparisons test: **P=0.0005 and ***P<0.0001, respectively). *N*=4.

To test whether VHA and CA activities promote photosynthesis by *C. xamachana* symbiotic algae, we measured dark respiration (*R*_D_) and net photosynthetic (*P*_N_) rates from bell tissue fragments incubated with specific inhibitors of VHA (ConcA) and CA (EZ). An inhibitor of PSII electron transport (DCMU) served as a positive control. We confirmed that the microsensor readings (in the absence of jellyfish tissue) were not affected by changes in illumination or by addition of drugs (Fig. S3). We also confirmed that DMSO (the vehicle for drug delivery) did not alter jellyfish *R*_D_ or *P*_N_ (Fig. S4). Moreover, none of the three drugs affected *R*_D_ (Fig. 6A). In contrast, VHA inhibition with ConcA significantly decreased *P*_N_ by 47±32% (range 13–100%) and CA inhibition with EZ significantly decreased *P*_N_ by 35±14% (range 10–50%) (Fig. 4B). As expected, PSII inhibition with DCMU completely ablated *P*_N_ (Fig. 6B). PAM fluorometry measurements revealed that only DCMU had a significant effect on *F*_v_/*F*_m_ or *Y*(II) (Fig. 7).

## DISCUSSION

Our results indicate that the symbiotic amoebocytes of *C. xamachana* contain abundant VHA and CA in close association with their symbiosomes, and that the activities of both enzymes contribute to a CCM that promotes photosynthesis by their endosymbiotic algae. These findings match results from coral and anemone symbiocytes (Barott et al., 2015, 2022). However, the independent evolution of endosymbiosis in scyphozoan jellyfish and anthozoans (Kayal et al., 2018) and the different cell type identity of amoebocytes and symbiocytes indicate a case of convergent evolution rooted in ancestral immunological and food digestion functions, respectively. Moreover, an analogous CCM in secondary endosymbiotic phytoplankton such as diatoms indicate the links between phagocytosis and CCM extend to single-celled organisms (Yee et al., 2023) and that they play critical roles in shaping the evolution of animal–microbe and microbe–microbe interactions.

In *C. xamachana* amoebocytes, VHA was closely associated with every symbiosome in a continuous ring-like pattern around each algal endosymbiont (Fig. 2). In contrast, many symbiocytes of corals and anemones display a punctate VHA signal around the symbiosome whereas others lack VHA_­_ entirely (Barott et al., 2015, 2022). The uniform association of VHA_B_ with amoebocyte acidic pH symbiosomes (Fig. 3) matches typical features of professional phagocytotic immune cells from both invertebrate and better-studied vertebrate animals (reviewed in Grinstein et al., 1992). The western blot results (Fig. 1) showed that tissue fractions enriched for symbiotic amoebocytes generally had greater VHA_B_ abundance that those lacking symbiotic amoebocytes; however, one of the four anemones tested had the opposite pattern. This could be due to temporal heterogeneity in the symbiotic process, in the concentration of symbiotic amoebocytes, or both between the jellyfish (and jellyfish tissue samples) we tested.

Amoebocytes also contained a ∼80 kDa CA-like protein (Fig. 4), which we believe corresponds to an α-class CA predicted from the *C. xamachana* genome (Ohdera et al., 2019). Although this predicted CA is ∼75 kDa, it contains two N-glycosylation sites that likely explain the ∼80 kDa band, and an extracellular signal peptide (Fig. S2) that suggests the mature protein is excreted out of the amoebocyte or into intracellular compartments. The presence of CA in amoebocytes has been previously reported (Estes et al., 2003) and our confocal Airyscan observations additionally show a close association of CA with the symbiosome. The extracellular signal peptide may imply that it faces the symbiosome space rather than the amoebocyte cytoplasm, but, unfortunately, our observations do not have sufficient resolution to discriminate between these two options. Interestingly, glycosylation can protect proteins from oxidative damage (reviewed in Gautam et al., 2020), which would be advantageous for this CA given its intimate proximity to O_2_-producing endosymbiotic algae.

Although previous studies have shown that the symbiocytes of anthozoans also have abundant CA in close proximity with the symbiosome (Weis, 1991, 1993; Weis et al., 1989), that protein seemed to be located in the cytoplasm and it was ∼30 kDa, suggesting a relationship with CA2 from vertebrates. In contrast, the *Cassiopea* CA in our study was similar in size, glycosylation potential and presence of an extracellular signal peptide to a CA from symbiotic tridacnid giant clams (Baillie and Yellowlees, 1998; Leggat et al., 2005). However, our results do not rule out the presence of CA2-like proteins in the amoebocyte cytoplasm or of CA4-like proteins in its cell membrane, and highlight the need for studies that establish the subcellular localization of proteins to better understand the mechanisms underlying the physiology of symbioses. For example, not all amoebocytes displayed CA immunoreactivity, which might reflect different symbiosome phagolysosomal maturation stages, DIC demand and photosynthate production rates of individual algal symbionts within an amoebocyte, or symbiosome dynamics in relation to algal division. Along the same lines, both VHA and CA were abundantly present in the apical region of *C. xamachana* epithelial cells, suggesting their participation in acid–base relevant fluxes between the animal and the external environment that may additionally contribute to CCM and algal photosynthesis. However, VHA and CA in these cells could be alternatively or additionally related to the robust endocytic activity that is typical of cnidarian epithelial cells (Ganot et al., 2020). Another important consideration in our study is that the antibodies we used are polyclonal and were raised against the entire human CA2 protein. This might explain the presence of multiple bands in our *C. xamachana* western blots (Fig. 4) owing to conservation of amino acid stretches among CA isoforms, especially in the catalytic domains. It is worth noting that the western blot banding pattern of *C. xamachana* tissue fractions enriched for symbiotic amoebocytes was different from that of tissue fraction lacking symbiotic amoebocytes, that the latter had intense bands around ∼30 kDa that matched the typical size of CA2s (Fig. 4), and that CA2s are cytosolic proteins that match the subcellular localization seen in the epidermal and gastrodermal cells (Fig. 5A,B).

Similar to anthozoans, pharmacological inhibition of VHA (Barott et al., 2015, 2022) or CA (Weis, 1993; Weis et al., 1989) significantly decreased *P*_N_ (Fig. 4B). Several lines of evidence give us confidence that these effects reflect the involvement of VHA and CA activities in a host-controlled CCM and are not off-target effects of the drugs. In our experiments, neither ConcA nor EZ affected *R*_D_ (Fig. 4A), ruling out generally toxic effects or a prominent role of VHA and CA in non-photosynthetic metabolic processes that consume O_2_. Moreover, symbiotic Symbiodiniaceae typically have much lower CA activity than their host cells (Graham et al., 2015; Weis et al., 1989), the IC_50_ of EZ for *Symbiodiniaceae* CA activity (∼170 μmol l^−1^; Al-Moghrabi et al., 1996) is much greater than the EZ concentration used in our study (20 μmol l^−1^), Symbiodiniaceae algae downregulate CCM-associated CAs *in hospite* (Mashini et al., 2023) and VHA inhibition did not impair photosynthetic O_2_ production by algae freshly isolated from coral (Barott et al., 2015). Finally, the ConcA and EZ concentrations we used did not significantly affect *F*_v_/*F*_m_ or *Y*(II) (Fig. 5), thus ruling out potential direct off-target effects on the algal photosystems. Altogether, the data indicate that VHA and CA activities in amoebocytes act to concentrate CO_2_ in the symbiosome space, which then fuels photosynthesis by the endosymbiotic algae.

The endosymbiotic algae within *Cassiopea* amoebocytes arguably require a more robust CCM than those within anthozoan symbiocytes. Amoebocytes host many more algal endosymbionts than symbiocytes, a crowding that exacerbates CO_2_ limitation owing to inter-algal competition. Additionally, the low gas permeability of the mesoglea (Brafield and Chapman, 1983) limits the rate of CO_2_ supply from other cells within the jellyfish and from seawater, and also results in O_2_ build-up (Arossa et al., 2021; Lyndby et al., 2023), and thus a robust CCM is thus essential to ensure CO_2_ can outcompete O_2_ for Rubisco binding. Despite these challenges, amoebocytes are able to deliver DIC to their algal endosymbionts at high enough rates to sustain efficient photosynthesis that can provide enough photosynthates to meet the jellyfish respiratory carbon demand and, provided enough nitrogen and phosphate, additionally support tissue growth (Welsh et al., 2009).

The acidic pH within the symbiosome (Fig. 2I,J) is likely to play multiple other roles in addition to that as a CCM. Specifically, it might promote the transport of ammonia and trapping of ammonium into the symbiosome as proposed for coral symbiocytes (Thies et al., 2022), induce the release of algal monosaccharides (Ishii et al., 2023) and regulate algal cell division (Tang, 2015). Moreover, the link between the amoebocyte VHA-powered CCM and the phagolysosomal machinery is compatible with classic studies showing that amoebocytes digest unhealthy and dead algal endosymbionts following phago-lysosome fusion (Colley and Trench, 1985; Fitt and Trench, 1983). And it may also provide a mechanistic explanation for a recent report suggesting that degradation and loss of algal endosymbionts in *Cassiopea* exposed to heat stress is, at least in part, due to digestion by amoebocytes (Toullec et al., 2024).

From a broader perspective, acidification is a key component of food digestion and pathogen defense mechanisms, both intracellularly in phagocytic cells and extracellularly within gut lumens. The conserved role of VHA and CAs in independently evolved intracellular CCM of secondary endosymbiotic phytoplankton (Yee et al., 2023), anthozoan cnidarians (Barott et al., 2015, 2022) and scyphozoan cnidarians (present study), as well as in the extracellular CCM of giant clams (Armstrong et al., 2018), provide additional links with animal–microbe symbioses through the creation of host-controlled microenvironments that promote algal photosynthesis. Moreover, VHA and CAs are conspicuous in diverse microbe-hosting epithelia, including the trophosomes of the deep-sea worms *Riftia* (De Cian et al., 2003) and *Osedax* (Tresguerres et al., 2013), the gills of *Bathymodiolus* deep-sea mussels (Tame et al., 2023) and the light organ of bobtail squid (Hargadon et al., 2024). Thus, VHA and CAs are likely to modulate photosynthetic, chemioautotrophic, heterotrophic and bioluminescent symbioses in marine invertebrates. Very recently, VHA was shown to also be the main acidifying mechanism of the hagfish gut lumen (Weihrauch et al., 2025); however, more derived vertebrates acidify their ‘true stomachs’ using the H^+^/K^+^-ATPase (HKA) that arose after the 2R genome duplication with gnathostomes (Castro et al., 2014). The lesser acidifying power of VHA (∼pH 4) compared with HKA (∼pH 1) and the relative efficiency of their associated digestive enzymes are poised to be key factors that differentially shape microbiomes, parasite pathogenic strategies, and mechanisms underlying nutrient absorption and immunity in invertebrate and vertebrate animals.

## Supplementary Material

10.1242/jexbio.249849_sup1 Supplementary information
