# Supplementary material for: Co-option of immune and digestive cellular machinery to support photosymbiosis in amoebocytes of the upside-down jellyfish Cassiopea xamachana
Source: J Exp Biol. 2025 May 12;228(14):jeb249849. doi: 10.1242/jeb.249849 (PMC12091945; doi:10.1242/jeb.249849)
Supplement: Supplementary information [file jexbio-228-249849-s1.pdf]

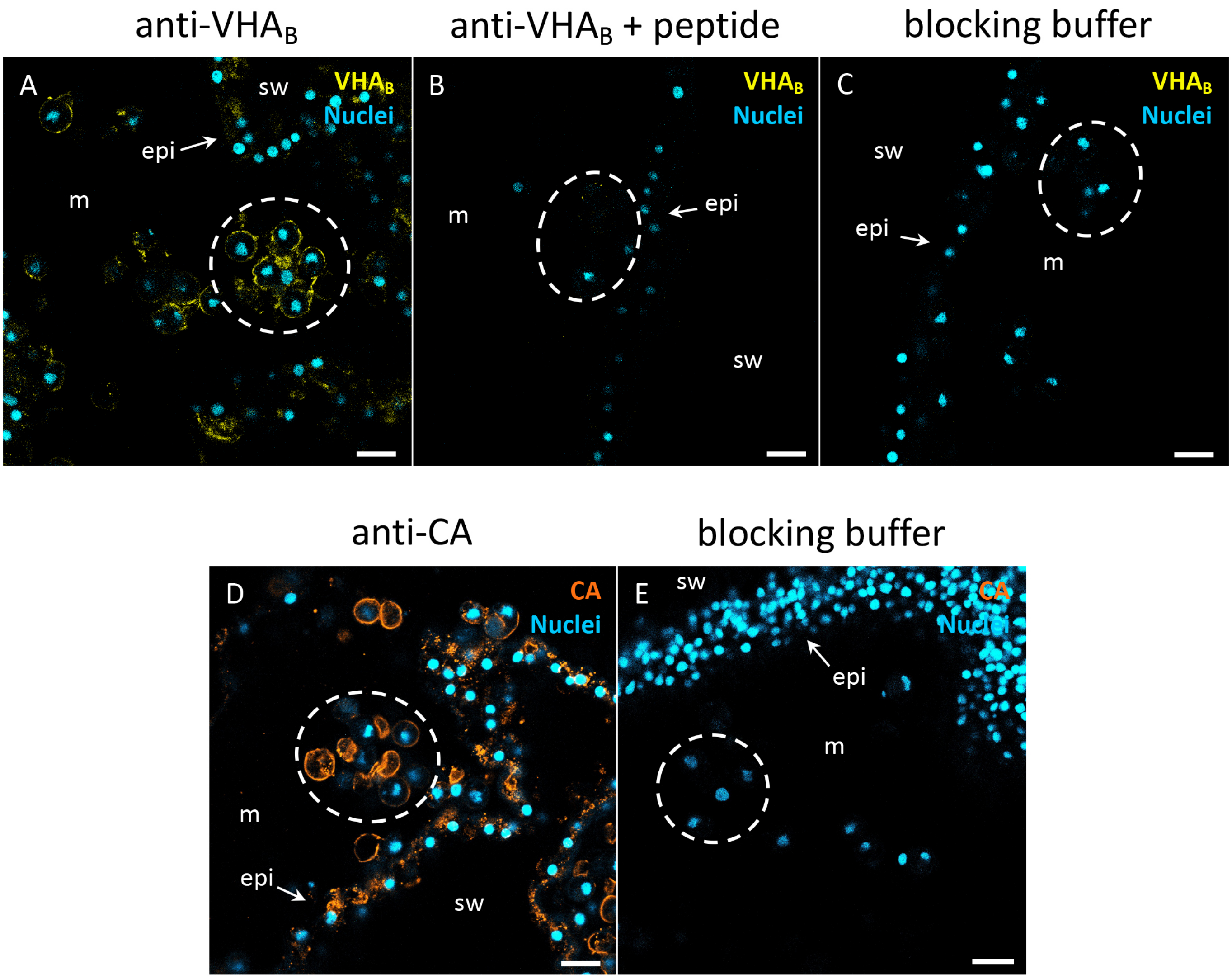

Fig. S1. Immunohistochemical validation of antibodies in *C. xamachana* amoebocytes.

(A) anti-VHA$_B$ antibodies. (B) anti-VHA$_B$ antibodies incubated with 13-fold excess peptide. (C) Blocking buffer only. (D) anti-CA antibodies. (E) Blocking buffer only. Amoebocytes are marked in dashed circles. All scale bars are 10 µm. epi-epidermis; m-mesoglea; sw-seawater.

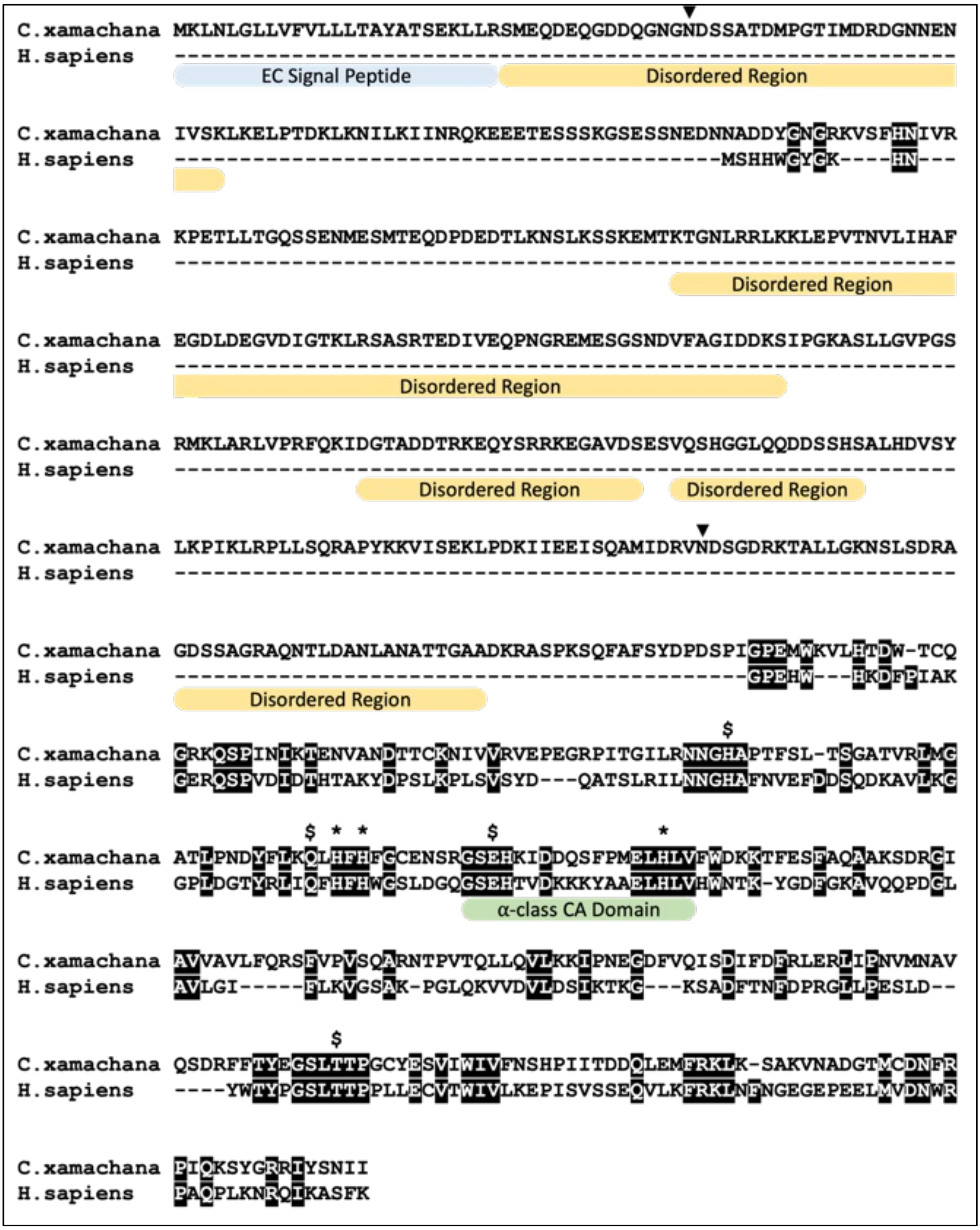

Fig. S2. Alignment of *Cassiopea xamachana* CA [Protein ID 1670; (Ohdera et al., 2019)] with *Homo sapiens* CAII (NP_000058.1). Conserved residues are highlighted in black (BoxShade). Extracellular signal peptide (EC Signal Peptide) as predicted by DeepLoc v2.0 (Thumuluri et al., 2022), disordered regions and α-class carbonic anhydrase domain predicted by InterPro v98.0 (Paysan-Lafosse et al., 2023), N-linked glycosylation sites (black triangles) predicted by NetNGlyc v1.0 (Gupta and Brunak, 2002) and conserved active sites ($) and zinc-binding domains (*) as discussed in (Supuran, 2008) are marked.

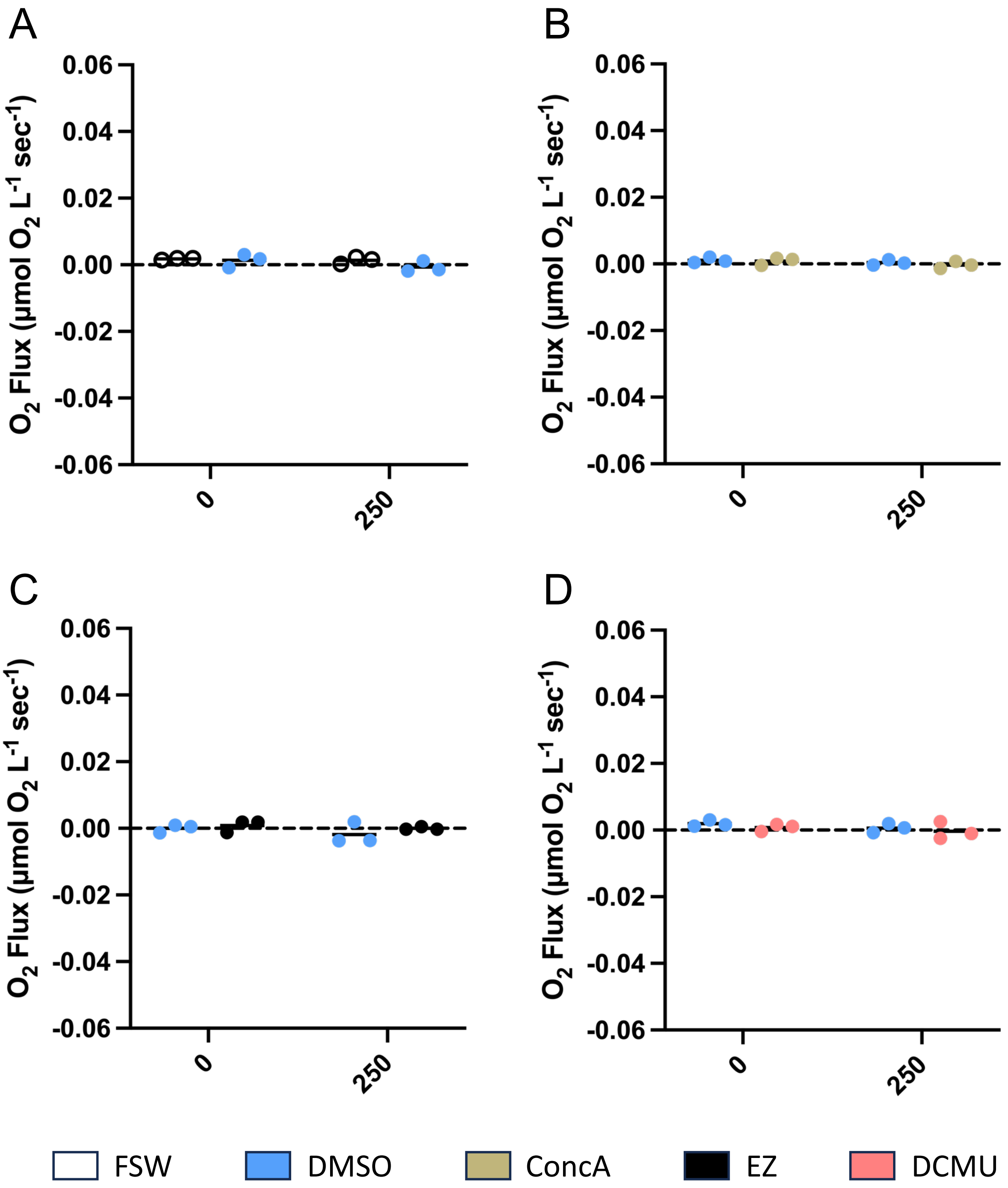

Fig. S3. Respirometry microsensor was not affected by light exposure nor drug treatment. Addition of (A) DMSO, (B) ConcA, (C) EZ, nor (D) DCMU significantly changed microelectrode readings in empty respirometry chambers (2-way ANOVA; $p > 0.05$). $N = 3$ for all treatments.

Journal of Experimental Biology • Supplementary information

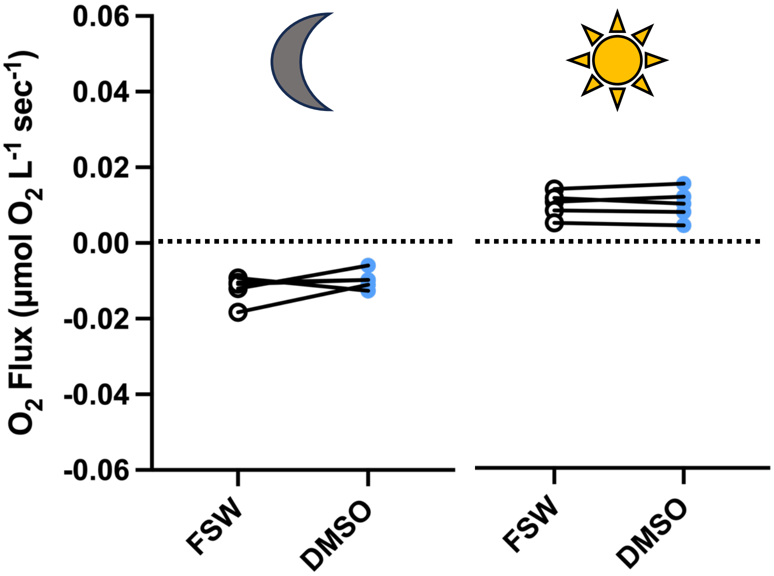

Fig. S4. Effect of DMSO on *C. xamachana* R_D and P_N. Bell tissue fragments were incubated with FSW then DMSO in darkness or under 250 µmol photons m^{-2} s^{-1}. DMSO treatment did not alter R_D nor P_N (paired t-tests; $p > 0.05$). N = 5 for all treatments.
